# Phase II study of capecitabine and irinotecan combination chemotherapy in patients with advanced gastric cancer

**DOI:** 10.1038/sj.bjc.6603093

**Published:** 2006-04-25

**Authors:** J H Baek, J G Kim, S B Jeon, Y S Chae, D H Kim, S K Sohn, K B Lee, Y J Choi, H J Shin, J S Chung, G J Cho, H Y Jung, W Yu

**Affiliations:** 1 Department of Oncology/Hematology, Kyungpook National University Hospital, Daegu, Korea; 2 Department of Oncology/Hematology, Pusan National University Hospital, Pusan, Korea;; 3 Department of General Surgery, Kyungpook National University Hospital, Daegu, Korea

**Keywords:** capecitabine, chemotherapy, gastric cancer, irinotecan

## Abstract

The present study was conducted to evaluate the efficacy and safety of a combination regimen of capecitabine plus irinotecan in patients with advanced gastric cancer. Patients with previously untreated metastatic or recurrent, measurable gastric cancer received oral capecitabine 1000 mg m^−2^ twice daily from day 1 to 14 and intravenous irinotecan 100 mg m^−2^ on days 1 and 8, based on a 3-week cycle. Forty-one patients were enrolled in the current study, among whom 38 were assessable for efficacy and 40 assessable for toxicity. Three complete responses and 16 partial responses were confirmed, giving an overall response rate of 46.3%. At a median follow-up of 269 days, the median time to progression and overall survival were 5.1 and 8.6 months, respectively. Grade 3/4 neutropenia occurred in four patients and grade 3 febrile neutropenia was observed in two patients. Grade 3 diarrhoea and grade 2 hand–foot syndrome occurred in six patients and eight patients, respectively. The combination of capecitabine and irinotecan was found to be well tolerated and effective in patients with advanced gastric cancer. Accordingly, this regimen can be regarded as one of first-line treatment options for advanced gastric cancer.

Despite a declining incidence in many developed countries, gastric cancer remains the second most common cancer-related death in the world (Pisani et al, 1999). Although the prognosis for advanced gastric cancer is poor, combination chemotherapy has improved the quality of life and overall survival compared with the best supportive care in several randomised studies (Murad et al, 1993; Glimelius et al, 1994; Pyrhonen et al, 1995). Among the various active chemotherapeutic agents, cisplatin-based combination chemotherapy has been most commonly used with a high response rate of 48–56% (Kim et al, 1993; Boku et al, 1999; Roth et al, 2000). Yet, notwithstanding its active anticancer effect in the treatment of advanced gastric cancer, cisplatin also induces nausea and vomiting in most patients (Boku et al, 1999; Roth et al, 2000). Plus, despite the development of new antiemetic agents, nausea and vomiting are still the main treatment-interrupting complications.

As such, oral fluoropyrimidine capecitabine (Xeloda®; Hoffmann-La Roche Inc., Nutley, NJ, USA) was rationally designed to preferentially generate 5-FU in tumour tissue and mimic continuous-infusion 5-FU. This tumour selectivity is achieved through exploiting the significantly higher activity of thymidine phosphorylase in many tumour tissues compared with healthy tissue (Miwa *et al*, 1998; Schuller *et al*, 2000). In addition, capecitabine has also exhibited antitumour activity with tolerable safety profiles when given as a monotherapy or in combination with cisplatin in patients with various other solid tumours as well as advanced gastric cancer (Kim *et al*, 2002; Ahn *et al*, 2003; Koizumi *et al*, 2003).

Meanwhile, irinotecan is a semisynthetic, water-soluble derivative of the plant alkaloid camptothecin. After conversion to its active metabolite, SN-38, irinotecan acts by inhibiting the enzyme DNA-topoisomerase I. Irinotecan has also shown promising activity in advanced gastric cancer as a single agent or combined with other agents (Boku *et al*, 1999; Ajani *et al*, 2001; Bugat, 2003).

The combination of capecitabine plus irinotecan has already shown synergistic antitumour activity in preclinical and clinical studies (Cassata *et al*, 2001; Jordan *et al*, 2002), where preclinical evidence indicated that irinotecan upregulates thymidine phosphorylase expression (Jordan *et al*, 2002), possibly providing the basis for the synergistic antitumour activity of the capecitabine and irinotecan combination. Furthermore, capecitabine and irinotecan have distinct action mechanisms and only partially overlapping adverse event profiles.

Although several studies have shown the efficacy and safety of capecitabine and irinotecan for advanced colorectal cancer (Tewes *et al*, 2003; Bajetta *et al*, 2004; Borner *et al*, 2005; Kim *et al*, 2005; Rea *et al*, 2005), no results have yet been reported for advanced gastric cancer. Accordingly, the current phase II study was conducted to evaluate the efficacy and safety of a combination regimen of capecitabine plus irinotecan in patients with advanced gastric cancer.

## PATIENTS AND METHODS

### Eligibility

All the patients involved in the current study had histologically confirmed metastatic or recurrent gastric adenocarcinoma with at least one unidimensionally measurable lesion. The patients were 18–75 years of age with a performance status of 0–2 on the Eastern Cooperative Oncology Group (ECOG) scale. Also, adequate haematological (absolute neutrophil count ⩾1.5 × 10^9^ l^−l^, platelet count ⩾100 × 10^9^ l^−l^, and haemoglobin ⩾9 g dl^−1^), renal (serum creatinine ⩽1.5 mg dl^−1^ and creatinine clearance ⩾50 ml min^−1^), and hepatic (total bilirubin ⩽1.5 mg dl^−1^ and serum transaminase level ⩽2.5 times the upper normal limit (UNL) or ⩽5 times the UNL in cases of hepatic metastases) functions were also required. Patients were ineligible if they had previously received palliative chemotherapy or radiation therapy, or had other severe medical illnesses, CNS metastasis, or another active malignancy. The protocol was approved by the institutional review board of each centre, and written informed consent obtained from all patients before enrolment. The ethical opinion was also considered by the institutional review board.

### Study treatment

All the treatments were administered on an outpatient basis. Capecitabine 1000 mg m^−2^ b.i.d. with pyridoxine 100 mg t.i.d. was given on days 1–14 followed by a 7-day rest period. The capecitabine was supplied as film-coated tablets at two dose strengths, 150 and 500 mg, whereas the irinotecan 100 mg m^−2^ was administered through a 90-min intravenous infusion on days 1 and 8, based on a 3-week cycle. All patients received 5-HT_3_ inhibitors for emesis prophylaxis. Treatment was continued until disease progression, patient refusal, or an unacceptable toxicity with a maximum of nine cycles.

### Dose modification

The next course of treatment only began when the neutrophil count was ⩾1.5 × 10^9^ l^−l^, the platelet count was ⩾75 × 10^9^ l^−l^, and any other treatment-related toxicities were less than or equal to grade 1; otherwise, treatment was withheld for up to 2 weeks. If adverse events did not improve to grade 0 or 1 after 3 weeks, the patients were excluded from the study.

The capecitabine treatment within a particular cycle was withheld in the presence of repeated grade 2 or any grade ⩾3 haematological or non-haematological toxicity. The capecitabine treatment was then resumed at a 25% dose reduction after a resolution of the toxicity to grade 0–1. An additional 25% dose reduction was also applied in the case of repeated grade ⩾3 toxicity. For grade 2–3 hand–foot syndrome (HFS), the capecitabine treatment was withheld until a resolution to less than or equal to grade 1, then restarted with a 25% dose reduction.

The irinotecan treatment on day 8 was omitted in the presence of grade ⩾3 haematological or non-haematological toxicity on the day scheduled for the irinotecan administration, and the patient then re-evaluated weekly until regressing to less than or equal to grade 1. Missed doses of irinotecan were not made up. The following cycle of treatment was reduced by 25% in the case of repeated grade 2 or any grade 3 toxicity and reduced by 50% in the case of repeated grade 3 or any grade 4 toxicity during the preceding cycle.

### Study assessments

A screening assessment, including the medical history, a physical examination, ECG, chest X-ray, and tumour assessment, was conducted within 2 weeks before starting the treatment. Further assessments conducted within 7 days before starting the treatment included vital signs, an ECOG performance status, and laboratory tests (haematology, blood chemistry, and urinalysis). Complete blood counts were performed before and on day 8 of each cycle, and biochemical tests performed before each cycle. The tumours were measured by computed tomography (CT) scans every three cycles until the tumour progressed. The tumour responses were classified according to the response evaluation criteria in solid tumours (RECIST) guidelines (Therasse *et al*, 2000) complete response (CR), the disappearance of all target lesions; partial response (PR), a decrease of at least 30% in the sum of the longest diameters of the target lesions; progressive disease (PD), an increase of at least 20% in the sum of the longest diameters of the target lesions or the appearance of one or more new lesions; stable disease (SD), neither sufficient shrinkage to qualify for PR nor a sufficient increase to qualify for PD. Complete response or PR patients were required to undergo a confirmatory disease assessment at least 4 weeks later. Adverse events were graded according to the National Cancer Institute Common Toxicity Criteria for Adverse Events (NCI-CTCAE) version 3.0; HFS was graded 1–3, as defined in previous capecitabine clinical studies (Blum *et al*, 1999).

### Statistical analysis

The current trial was designed to detect a response rate of 40% as compared to a minimal, clinically meaningful response rate of 20%. Plus, the current trial used a two-stage optimal MiniMax design, as proposed by Simon, (1989), with an 80% power to accept the hypothesis and 5% significance to reject the hypothesis (Simon, 1989). Allowing for a follow-up loss rate of 10%, the total sample size was 37 patients with a measurable disease. The duration of response, time to progression (TTP), and survival analysis were estimated using the Kaplan–Meier method. The duration of response was defined as the interval from the onset of CR or PR until evidence of PD was found. Meanwhile, the time to progression was calculated from the initiation of chemotherapy to the date of disease progression, and the overall survival measured from the initiation of chemotherapy to the date of the last follow-up or death. The statistical data were obtained using an SPSS software package (SPSS 11.5 Inc., Chicago, IL, USA).

## RESULTS

### Patient characteristics

Between July 2004 and March 2005, 41 patients were enrolled in the present study from two centres. The characteristics of the patients are summarised in Table 1. The median age was 59.0 (range: 25–74) years, and there were 30 men and 11 women. Most of the patients (92.7%) had a good performance status (ECOG 0 or 1). Twenty-nine (70.7%) patients were suffering from a metastatic disease, whereas 12 patients had a recurrent disease after surgical resection (total or subtotal gastrectomy) of the primary tumour. Distal lymph nodes and the liver were the most common sites of the metastases.

### Efficacy

**Figure 1:**
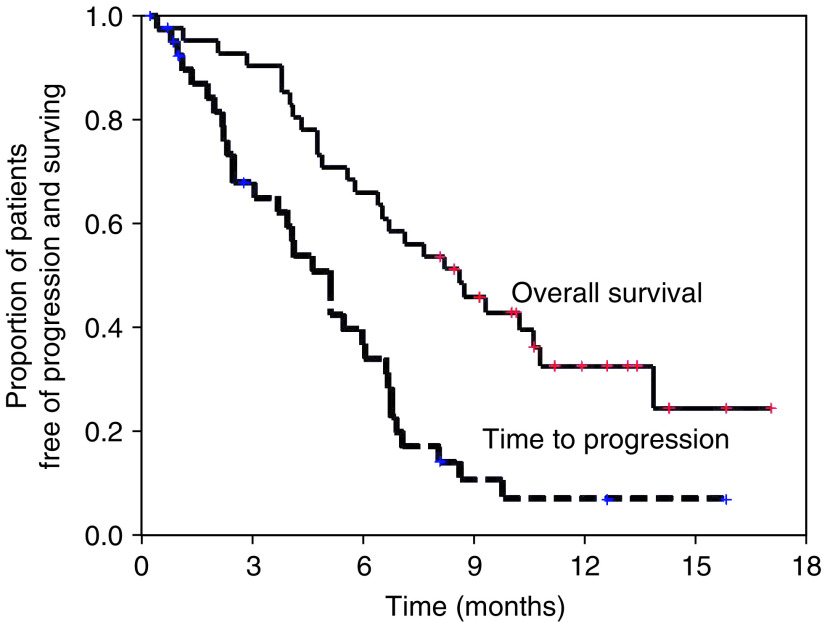
Kaplan–Meier curves for time to disease progression and overall survival for intention-to-treat population (*n*=41).

Thirty-eight of the 41 patients (92.7%) were assessable for response, with the remaining three being lost to follow-up or patient refusal. All efficacy data are reported using the intention-to-treat patient population. Three cases of CR and 16 cases of PR were confirmed, giving an overall response rate of 46.3% (95% CI: 30.4–62.3%). The response characteristics are shown in Table 2. The median duration of response in the 19 responding patients was 4.6 months (95% CI: 3.3–5.9 months), whereas the median TTP for all patients was 5.1 months (95% CI: 3.9–6.3 months) at a median follow-up duration of 269 days (range: 13–519 days). Twenty-seven patients had died at the time of the present evaluation. The median overall survival was 8.6 months (95% CI: 6.1–11.1 months) with a 1-year survival rate of 32.6% (Figure 1).

### Toxicity

The haematological and non-haematological toxicities that occurred during the current study are summarised in Table 3. A total of 160 cycles (median 3, range 1–9 cycles) were administrated in 40 patients assessable for toxicity. The most severe haematological adverse event was neutropenia, which occurred with a grade 3/4 intensity in four patients (10.0%) in seven cycles (4.4%). Plus, febrile neutropenia was observed in two patients (5.0%). All cases were successfully treated with antibiotics and G-CSF, and there were no treatment-related deaths during this study. The most common non-haematological toxicity was nausea (grade 1/2, 80.0%). Grade 3 diarrhoea occurred in six patients (15.0%) and grade 2 HFS, a complication of capecitabine, occurred in four patients (10.0%). Yet, no grade 4 non-haematological toxicity was observed. Overall, 10 (25.0%) patients and 27 (16.9%) cycles required a dose reduction of irinotecan on day 1, whereas dose omissions of irinotecan on day 8 were needed in 18 (11.3%) cycles. Also, a total of eight (5.0%) cycles were delayed. The most common reasons for the dose modification of irinotecan were neutropenia (eight patients, 13 cycles) and diarrhoea (nine patients, 11 cycles). Meanwhile, the capecitabine doses were modified mainly owing to neutropenia, diarrhoea, or HFS. The mean dose intensity over all the treatment cycles was 8641 mg m^−2^ week^−1^ for capecitabine and 58.4 mg m^−2^ week^−1^ for irinotecan, corresponding to 92.6 and 87.6% of the planned dose intensity, respectively. The compliance with capecitabine was 96.0% for all the treatment cycles.

## DISCUSSION

In the current study, the combination chemotherapy of capecitabine and irinotecan, which can be administered on an outpatient basis, produced active antitumour activity and a safe toxicity profile in patients with advanced gastric cancer. The overall response rate (46.3%), median TTP (5.1 months), and median overall survival (8.6 months) following treatment with the present regimen were comparable with previous results reported for cisplatin-based combinations (Kim *et al*, 1993; Boku *et al*, 1999; Roth *et al*, 2000; Vanhoefer *et al*, 2001), where a continuous infusion of a 5-FU and cisplatin regimen achieved a response rate of 37–51% and median overall survival of 9–9.7 months (Kim *et al*, 1993; Vanhoefer *et al*, 2001), whereas docetaxel or irinotecan plus cisplatin regimens achieved a response rate and median overall survival of 48–56% and 9–9.06 months, respectively (Boku *et al*, 1999; Roth *et al*, 2000).

Recently, in meta-analysis for advanced gastric cancer (Wagner *et al*, 2005), combination chemotherapy improved survival compared to single-agent 5-FU, and best survival results were achieved with regimens containing 5-FU, anthracycline, and cisplatin among the various combination chemotherapies. However, Dank *et al* (2005) reported that irinotecan plus 5-FU/folinic acid showed a trend to TTP superiority, compared with cisplatin and 5-FU, as well as a better safety profile in a randomised phase III trial. And they suggested that irinotecan plus 5-FU/folinic acid could be an alternative first-line treatment option without cisplatin for patients with advanced gastric cancer.

Capecitabine has already been shown to be active and safe in the treatment of previously untreated advanced gastric cancer. A recent phase II study by Kim *et al* (2002) reported that a capecitabine plus cisplatin regimen produced a high response rate of 54.8% and median overall survival of 10.1 months in patients with advanced gastric cancer. In contrast, the current study used a reduced dose of capecitabine, 1000 mg m^−2^ instead of 1250 mg m^−2^, owing to the relatively high incidence of HFS. Grade 2/3 HFS has previously been observed in 27.5–50% of patients with advanced gastric cancer who received the standard dose of capecitabine (Kim *et al*, 2002; Park *et al*, 2004), and as there is no effective prophylaxis or treatment for HFS, this can interrupt treatment or reduce the dose intensity of capecitabine. In the present trial, only four patients (10.0%) experienced grade 2 HFS, allowing the dose intensity of capecitabine to reach 92.6%.

The major toxicities related to irinotecan are diarrhoea and myelosuppression, which are known to be dose-dependent. Chemotherapy-induced severe diarrhoea or neutropenia can also result in treatment-related hospitalisation or mortality, thereby compromising the quality of life and increasing medical expenditure. Ajani *et al* (2002) reported that the weekly administration of irinotecan (65 mg m^−2^) and cisplatin (30 mg m^−2^) for 4 consecutive weeks followed by a 2-week break showed active antitumour activity in patients with untreated, advanced adenocarcinoma of the stomach or gastroesophageal junction. However, in their study, 27% of the patients experienced grade 3/4 neutropenia and 22% experienced grade 3/4 diarrhoea. Thus, owing to the high incidence of toxicities and treatment interruptions, they suggested that modification of the doses and schedule might be warranted to make the regimen more tolerable to patients. The combination regimen of irinotecan (80 mg m^−2^) followed by folinic acid (500 mg m^−2^) and 5-FU (2000 mg m^−2^ i.v. over 22 h) weekly for 6 weeks also showed 25% of grade 3/4 neutropenia, 5% of febrile neutropenia, and 22% of grade 3/4 diarrhoea in a randomised trial (Dank *et al*, 2005).

Recently, in a randomised multicentre phase II trial (Bajetta *et al*, 2004) comparing two different schedules of irinotecan combined with capecitabine as the first-line treatment for metastatic colorectal cancer, diarrhoea, which occurred in 37.8% of the patients at a grade 3/4 intensity, was the main adverse effect of the arm B regimen (capecitabine 1000 mg m^−2^ twice daily on days 2–15 and irinotecan 120 mg m^−2^ on days 1 and 8, every 21 days). However, in the present study, a reduced dose of irinotecan (100 mg m^−2^ on days 1 and 8) was administered to alleviate adverse effects, and grade 3/4 diarrhoea and neutropenia was only observed in 15 and 10% of the patients, respectively. Furthermore, there was no treatment-related death or grade 4 non-haematological adverse event.

In case of an irinotecan and 5-FU combination, the schedule-dependent interactions with respect to toxicity have already been demonstrated. Thus, a schedule of irinotecan followed by 5-FU infusion was found to be less toxic than the reversed schedule, and explained by a reduced SN-38 area under the curve level when the irinotecan preceded the infusional 5-FU (Falcone *et al*, 2001). Accordingly, in the present study, the irinotecan infusion preceded the capecitabine medication. As such, the better tolerability of the present regimen may have been associated with the schedule-dependent interaction between capecitabine and irinotecan.

In conclusion, the combination of capecitabine and irinotecan was found to be well tolerated and effective in patients with advanced gastric cancer. Accordingly, this regimen can be regarded as one of first-line treatment options for advanced gastric cancer.

## Figures and Tables

**Table 1:** Patient characteristics

**Characteristic**	**Number of patients (*N*=41, %)**
*Age (years)*	
Median	59
Range	25–74
	
*Gender*	
Male	30 (73.2)
Female	11 (26.8)
	
*ECOG performance status*	
0	1 (2.4)
1	37 (90.2)
2	3 (7.3)
	
*Disease status*	
Metastatic	29 (70.7)
Recurrent	12 (29.3)
	
*Location of primary tumour*	
Upper	8 (19.5)
Middle	11 (26.8)
Lower	22 (53.7)
	
*Histology*	
Adenocarcinoma, well differentiated	3 (7.3)
Adenocarcinoma, moderately differentiated	13 (31.7)
Adenocarcinoma, poorly differentiated	25 (61.0)
	
*Metastatic sites*	
Liver	21 (32.8)
Peritoneum	10 (15.6)
Distal lymph nodes	22 (34.4)
Ovary	6 (9.4)
Others (lung, bone, uterus, and spleen)	5 (7.8)
	
*Number of metastatic sites*	
1	21 (51.2)
2	19 (46.3)
⩾3	1 (2.4)

**Table 2:** Tumour response (intention-to-treat analysis)

**Response**	**Number (*n*=41, %)**
Confirmed response	19 (46.3)^a^
Complete response	3 (7.3)
Partial response	16 (39.0)
Stable disease	6 (14.6)
Progressive disease	13 (31.7)
Not assessable	3 (7.3)

a 95% CI=30.4–62.3%.

**Table 3:** Adverse reactions

	**Grade^a^ (% of patients, *n*=40)**				**Grade^a^ (% of cycles, *n*=160)**			
	**1**	**2**	**3**	**4**	**1**	**2**	**3**	**4**
*Haematological*								
Anaemia	35.0	30.0	2.5		30.0	11.3	1.3	
Leukopenia	10.0	22.5	5.0	2.5	13.1	10.6	2.5	0.6
Neutropenia	12.5	27.5	7.5	2.5	15.0	12.5	3.8	0.6
Thrombocytopenia	5.0				0.6			
								
*Non-haematological*								
Anorexia	22.5	17.5	2.5		10.6	6.3	0.6	
Nausea	60.0	20.0	2.5		25.0	9.4	0.6	
Vomiting	15.0	30.0	5.0		11.9	9.4	1.9	
Stomatitis	25.0	10.0	2.5		10.6	8.1	0.6	
Diarrhoea	22.5	20.0	15.0		11.3	6.9	5.0	
Constipation	10.0	2.5			3.8	0.6		
Abdominal pain	15.0	2.5			5.6	0.6		
Hand-foot syndrome	30.0	10.0			22.5	6.9		
Neuropathy	2.5	2.5			1.9	0.6		
Febrile neutropenia			5.0				1.3	
Infection without neutropenia			2.5				0.6	

a NCI-CTCAE v3.0, except for grading of hand–foot syndrome.
